# Study on GAP Adhesive-Based Polymer Films, Energetic Polymer Composites and Application

**DOI:** 10.3390/polym15061538

**Published:** 2023-03-20

**Authors:** Siyuan Wu, Xiaomeng Li, Zhen Ge, Yunjun Luo

**Affiliations:** School of Materials Science and Engineering, Beijing Institute of Technology, Beijing 100081, China

**Keywords:** adhesive, polymer film, energetic polymer composites, mechanical properties

## Abstract

To lay the foundation for environmentally friendly energetic polymer composites, GAP (glycidyl azide polymer) adhesive-based polymer films with different curing parameter R (mol ratio of hydroxyl/isocyanate) and energetic polymer composites with different RDX contents were studied. GAP/TDI (toluene diisocyanate)/GLY(glycerol) was selected as the adhesive system. The tensile strength and elongation at the break of the polymer film with R = 2.2, was 14.34 MPa and 176.86%, respectively, as observed by an AGS-J electronic universal testing machine. A relatively complete cross-linking network and high hydrogen bonding interaction were observed by LF-NMR (low-field nuclear magnetic resonance, where the cross-linking density was 11.06 × 10^−4^ mol/cm^3^) and FT-IR (fourier transform infrared spectroscopy, where the carbonyl bonding ratio was 64.84%). Forty percent RDX(hexogen) was added into the adhesive system. The tensile strength was 4.65 MPa, and the elongation at the break was 78.49%; meanwhile, the heat of the explosive was 2.87 MJ/kg, and the residue carbon rate was only 2.47%. The tensile cross-sections of energetic polymer composites were observed by SEM (Scanning electron microscopy).

## 1. Introduction

Energetic polymer composites, including solid propellant, polymer bonded explosive, gun propellants, and combustible cartridges, have been widely used as the power and damaged sources of missiles, torpedoes, guns, and explosives in the military weapon system due to their ultrahigh energy density and strong capacity for doing damage. However, when it comes to most of the energetic polymer composites, their polymer adhesives are not only non-energetic, but they also have low mechanical properties, leading to low energy density as well as mechanical properties [1].

GAP (glycidyl azide polymer), as an energetic adhesive, has the advantages of good burnout, high energy, high burning rate, and low sensitivity. N_2_ and CO_2_ are released after combustion, both of which are clean gases [2,3,4,5]. Therefore, it is very promising to prepare environmentally friendly energetic polymer composites [6]. Combustible cartridges, for example, are a kind of cartridge that can burn and provide energy. They have the advantages of being clean, light weight, providing energy supplies, not needing recycling like metal cartridges, reducing environmental pollution, and saving manpower and material costs [7,8,9]. It is a sustainable and environmentally friendly polymer. Therefore, we studied the use of the GAP adhesive system to prepare combustible components, such as combustible cartridges, and the design of the pouring process. However, GAP has poor mechanical properties due to the high steric hindrance of the azide group and the low number of atoms carried by the main chain, which is the common difficulty of GAP-based products, limiting the widespread use of GAP [10,11]. Researchers have explored several methods of overcoming this problem, such as adjusting the curing parameters, optimizing the cross-linking system, finding a suitable composite adhesive system, and adding reinforcing materials, etc. [9,12,13,14,15,16]. In this work, TDI was used as a curing agent, and glycerol was used as an adhesive. Additionally, we used glycerol as the three-functional cross-linking agent in the adhesive system, with a significant increase in the amount of glycerol, an increase in the cross-linking density of the system and a decrease in the molecular weight between the cross-linking points. The mechanical properties of GAP polymer film were optimized by changing the amount of glycerol and curing parameters. Subsequently, RDX was added to study the effects of the relative ratio of the adhesive system of RDX on the mechanical properties, the heat of the explosive, and the residue carbon rate. In this paper, the adhesive system was used to prepare combustible components such as combustible cartridges and cartridge boxes by the pouring process.

## 2. Materials and Methods

### 2.1. Materials

GAP (industrial grade, Mn = 3700) and dioctyl sebacate (DOS, industrial grade) were from the Luoyang Liming Chemical Research Institute (Luoyang, China). The GAP was dewatered in a vacuum drying oven at 80 °C for 24 h before use; glycerol (GLY, AR) was from Saran Chemical Technology Co., Ltd., Shanghai, China); toluene diisocyanate (TDI, industrial grade) was from Tianjin Guanghua Fine Chemical Research Institute (Tianjin, China); antifoaming agent (B-160, industrial grade) was from Guangdong Zhonglianbang Fine Chemical Co., Ltd., Guangdong, China; Dibutyltin dilaurate (DBTDL, AR) and triphenyl bismuth (TPB, AR) were from Beijing Chemical Plant (Beijing, China); Hexogen particles (RDX, 108 μm) were purchased from the North Huian Chemical Co., Ltd., Xi’an, China.

### 2.2. Preparation of Polymer Films

**Figure 1:**
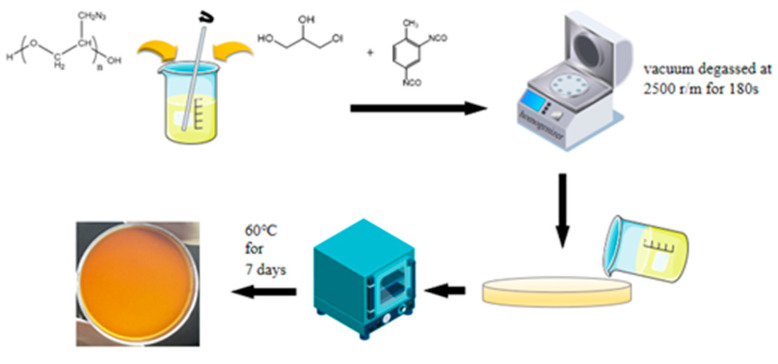
Schematic illustration for the preparation process of polymer films.

GAP and GLY were added to the beaker and stirred well. TDI, B-160, DBTDL and TPB (DBTDL: TPB = 1:3) were added to the above solution and mixed by a homogenizer keeping vacuum at 2500 r/m for 180 s to remove bubbles. The solution obtained was cast into a petri dish to cure for 7 days at 60 °C, as shown in Figure 1. In addition, the feeding ratio of all samples is shown in Table 1.

### 2.3. Preparation of Energetic Polymer Composites

RDX particles were added into the polymer matrix to prepare energetic polymer composite films, after determining the curing parameter (R, mol ratio of hydroxyl/isocyanate). Other steps were the same as above. The ambient humidity was 30% during the sample preparation process, because the ambient humidity can affect the sample curing if it is too high.

When the R = 1.0, the polymer film was not fully cured at 60 °C for 10 days; when the R = 2.6, although the sample could be cured at 60 °C for 1 day, the surface of the polymer film was uneven after curing because the sample was more sensitive to water. As a result, two samples with R = 1.0 and 2.6 could not be tested. Therefore, they will not be further discussed in the remainder of this text.

### 2.4. Characterization

The hardness of samples was tested by an LXD-A of the digital Shore A hardness tester (Shanghai Siwei Instrument Manufacturing Co., Ltd., Shanghai, China) at 25 °C. The chemical structure of samples was tested by Fourier transform infrared spectroscopy (FT-IR) by Nicolet 8700 infrared spectrometer (Thermo Fisher Scientific, Waltham, MA, USA). Test conditions: the test temperature was 25 °C, the number of scans was 32 times, the resolution was 4 cm^−1^, and the scanning range was 500–4000 cm^−1^. Static mechanical performance of samples was tested by an AGS-J electronic universal testing machine (Shimadzu Corporation, Japan), according to the method of GB/T528-1998. The test temperature was 25 °C, and the tensile rate was 100 mm/min. Low-field nuclear magnetic resonance (LF-NMR) of samples was tested by a VTMR20-010V-T nuclear magnetic resonance analyzer (Suzhou Niumai Technology Co., Ltd., Suzhou, China). Test conditions: The test temperature was 25 °C, and the number of accumulation times was 3. The heat of explosiveness of the samples was tested by an American Parr Company Parr6200 oxygen bomb calorimeter for testing, according to the GJB770B2005 gunpowder test method 701.1. The residue carbon rate was calculated by the proportion of the residual mass of the heat of the explosive sample. Scanning electron microscopy (SEM) was tested by A Hitachi S4800, Tokyo, Japan. Test conditions: acceleration voltage was 15 kv.

## 3. Results and Discussion

### 3.1. FT-IR of Polymer Films with Different R

**Figure 2:**
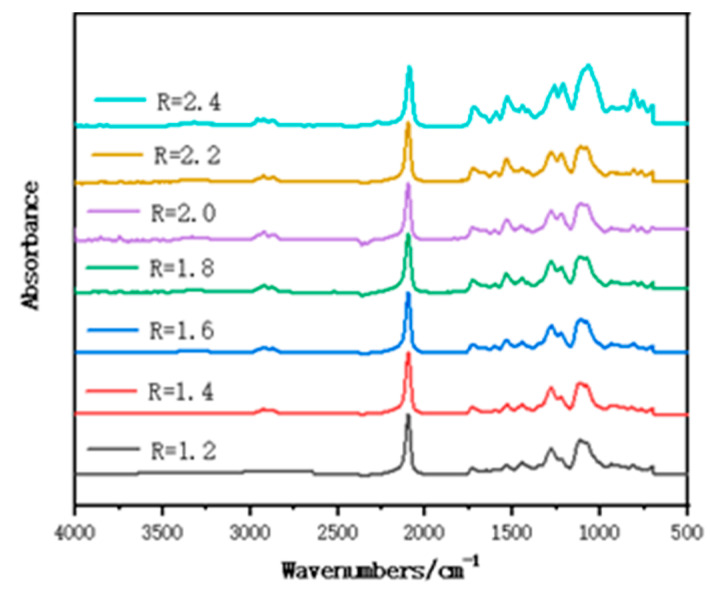
FT-IR spectrum of polymer films with different R.

Figure 2 shows the FT-IR spectrum of polymer films with different R; 2271 cm^−1^ and 2927 cm^−1^ are the characteristic absorption peaks of -NCO and C-H, and there is no obvious change with the increase in R, which indicates that both polyols and isocyanates have participated in the reaction; 2098 cm^−1^ is the -N_3_ characteristic absorption peak of GAP; 3346 cm^−1^ is the N-H stretching vibration peak; 2919 cm^−1^ is the C-H asymmetric stretching vibration peak; 2864 cm^−1^ is the C-H symmetric stretching vibration peak; 1716 cm^−1^, 1538 cm^−1^, and 1279 cm^−1^ are the characteristic absorption peaks of amide I, II and III bands on carbamate; 1446 cm^−1^ is the -CH_2_ bending vibration; 1353 cm^−1^ is the superposition of -CH bending vibration absorption peak; and -CN stretching vibration, the stretching vibration peak of C-O-C ether bond, is at 1109 cm^−1^, which indicates that carbamate group was successfully synthesized and GAP polymer films were successfully prepared. The full FT-IR spectrum of the samples proves that the sample has been successfully synthesized.

**Figure 3:**
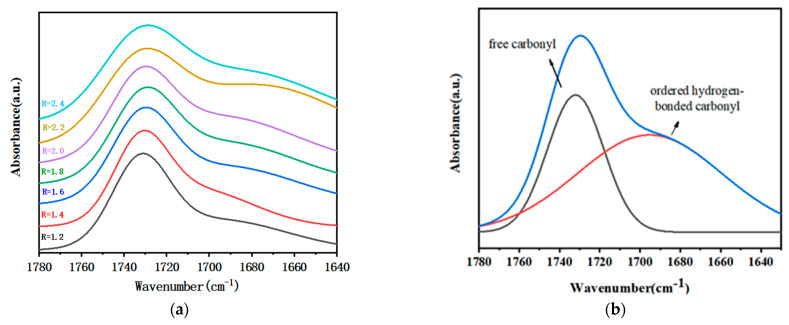
(**a**) FT-IR spectrum of carbonyl group of polymer films with different R; (**b**) FT-IR peak fitting spectrum of carbonyl group of polymer films.

Figure 3a shows the FT-IR spectrum of the carbonyl group of the polymer film with different R. To accurately analyze the hydrogen bond interaction of the polymer films, the second-order derivative spectrum was used to quantitatively identify the secondary structure of the carbonyl group; 1730 cm^−1^ and 1690 cm^−1^ were internal for the characteristic absorption peaks of free carbonyl and bonded carbonyl (Figure 3b). Gaussian function was used to fit the peak of the spectrum. After obtaining each sub-area, the ratio of its bonded carbonyl was calculated [17], and the calculated results are shown in Table 2.

Due to the formation of hydrogen bonds, the carbonyl peaks are split, and the FT-IR absorption peak positions of carbonyl groups that are involved in the formation of hydrogen bonds shift to lower wavenumbers. Thermodynamically, the structural units of the hard segment and the soft segment of the polymer films are incompatible or incompletely compatible, and the hard segments would aggregate with each other to produce dispersed micro domains and main micro phase separation. Micro phase separation not only played a role similar to filler reinforcement (inhibiting crack propagation), but also acted as a physical crosslink point for molecular chains, which is beneficial to improve the flammable cartridge mechanical properties. The hydrogen bonding between the hard segments in the polymer film is stronger, leading to a higher degree of micro phase separation.

The peak areas of free and bonded carbonyl groups are obtained from the sub-peaks, and the proportion of bonded carbonyl groups is shown in Table 2, which indicates that the proportion of bonded carbonyl groups of GAP/TDI/GLY polymer films increased first and then decreased, with the increase of R and the degree of micro phase separation also showing the same trend. The reason for that is: When the R is increased, the hard segments aggregate to form hard segment micro domains, and the degree of micro phase separation of the polymer film is enhanced due to the enhancement of hydrogen bonding between the hard segments. When R = 2.4, the mobility of the hard segment is restricted due to the high crosslink density in the polymer films, and the difficulty of formation of hydrogen bonds increases, resulting in a decrease in the degree of hard segment aggregation.

### 3.2. Crosslink Density of Polymer Films with Different R

The crosslinking density of polymer films was tested by low-field nuclear magnetic resonance (LF-NMR) to analyze the reason for enhancing mechanical tensile. As shown in Table 3, it was found that the crosslink density (ν_e_) of the polymer film increased with the increase of R. This is mainly attributed to the following two factors: on the one hand, -NCO can react with the urethane group and form a chemical crosslinking point; on the other hand, more hard segments increase the degree of micro phase separation; these hard segments are also viewed as physical crosslinking points. The increase in crosslinking density can restrict the mobility of molecule chains, leading to an increase in mechanical tensile.

The mechanical property of the polymer films is also related to the crosslink network structure. The actual crosslink network of the polymer film is not ideal, and there are other structural features [18,19] that are divided into the following cases: (1) the two ends of the same molecular chain are bonded to form a sealed circle; (2) only one end of the molecular chain enters the network structure, forming terminal defects; (3) temporary physical entanglement of molecular chains; (4) some groups between molecular chains form physical crosslinks due to hydrogen bonding. For these four cases, since quantitative calculation and statistics are not available, a correction factor (A) is introduced to represent the total contribution of these four effects to the mechanical properties of the crosslink network. Among the four cases, the former two cases cause defects in the crosslink network structure, resulting in a decrease in A, while the latter two cases will compensate for the elastic modulus of the crosslink network when subjected to external force, resulting in a larger A [17]. If A < 0, meaning that there are many network defects in the system; If A = 0, the compensation of elastic modulus by physical crosslink can offset the defects in the system; A > 0 means that the physical crosslink effect in the system is strong, and the crosslink network structure is more complete.

To study the crosslink network structure of the polymer film with different R, the relative molecular mass between the crosslink points (M_c_) was calculated by the Formula (1). According to Formula (2), we know that A is related to the shear modulus of samples. To calculate the shear modulus of samples, we first tested the elastic modulus of all samples and then calculated the shear modulus based on a relationship between elastic modulus and shear modulus (Formula (3)), when the strain of crosslink elastomer is very small. Based on experimental results and formulas, the correction factor (A) of all samples is shown in Table 3. As shown in Table 3, when R = 1.2–1.8, the A of the polymer film is negative, indicating that the system had many network structural defects, and the physical crosslink could not offset the negative effects of the network defects; when R = 2.0–2.4, the content of isocyanate groups increased in the system, the proportion of hydrogen bonds between molecular chains increased, and the probability of forming a cyclic structure also increased. These two interactions result in a gradual increase in the A value of the polymer films, implying that the physical crosslink effect is strong, and the structural integrity of the crosslink network is good. Therefore, with the increase of R, the cross-linking network structure of the polymer films was more complete, leading to an increase in tensile strength. In summary, the mechanical property of the polymer film is increased, which is due to high crosslink density and a more integrated molecular network structure.

(1) $$\nu_{e}=\frac{\left(ln(1-\nu_{2})+\nu_{2}+\chi \nu_{2}^{2}\right)}{\nu (\nu_{2}^{1/3}-2\nu_{2}/f)}=\frac{\rho_{b}}{M_{c}}$$

(2) $$G=\rho RT/M_{c}+A$$

(3) $$E=3\rho RT/M_{c}+3A$$

where *ν_e_* is the crosslink density of the elastomer; *ν*_2_ is the volume fraction of the rubber phase in the swollen elastomer; *ν* is the molar volume of the solvent; *χ* is the interaction parameter between the adhesive and the solvent, and *f* is the adhesive network The functionality of; *ρ_b_* is the density of the elastomer, *M_c_* is the average molecular mass between the crosslink points; *G* is the shear modulus, *E* is the elastic modulus, *M_c_* is the average molecular mass between the crosslink points, *A* is the correction factor, *R* is the thermodynamic constant, *T* is the temperature, *ρ* is the density.

### 3.3. Mechanical Property of Polymer Films with Different R

**Figure 4:**
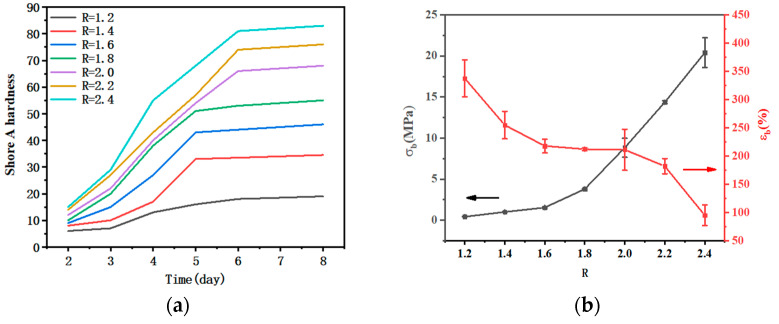
(**a**) Shore A hardness of the polymer films with different R; (**b**) curves of tensile strength and elongation at break of polymer films with different R.

As a characterization of resistance to deformation, Shore A hardness can also be used to describe the curing degree of the curing system and measure whether the curing is complete. As shown in Figure 4a, other GAP/TDI/GLY polymer films can form a regular shape after curing for 48 h, and the hardness of the polymer films remained unchanged after curing for six days, indicating that the polymer film is completely cured. In the case of large-scale production, the samples could be demoulded after curing for 48 h and waiting for complete curing.

As shown in Figure 4b, the tensile strength of polymer films increased with the increase of R, which is consistent with the results obtained from the proportion of infrared bonding. Due to the increase of R, the content of isocyanate and glycerol increased, and the content of hard segment and the cross-linking point in the polymer film increased, and the strength of the polymer film increased. However, as a three-functional cross-linking agent, the increase of the amount of glycerol significantly increased the cross-linking density of the system and reduced the molecular weight between the cross-linking points, resulting in the molecular chain being difficult to fully extend freely due to the increase of cross-linking density during the stretching process, while elongation at the break decreased. When R = 1.2–2.4, the tensile strength of polymer films increased from 0.43 MPa to 19.58 MPa, and the elongation at the break decreased from 337.51% to 94.98%, respectively. The higher tensile strength was better, yet a lower strength could be accepted if the elongation at break exceeds 30%. This is because the requirement of the propellant under normal circumstances is that the elongation at the break is higher than 30%. Since there is no specific requirement for the combustible element, this paper refers to the requirements of the propellant, considering that the tensile strength of polymer films would decrease after adding RDX. Therefore, herein we choose the range of R = 2.0, 2.2 and 2.4, the tensile strength of the polymer film is increased from 8.83 MPa to 19.58 MPa, and the elongation at the break decreased from 211.29% to 94.98%, respectively. Based on the above results, we find that when R = 2.0, 2.2, 2.4, the mechanical properties of samples are optimum. However, the polymer film with a higher R is more sensitive to water. Therefore, when R = 2.2, the polymer film is chosen and used as a polymer adhesive in energetic polymer composites in the next text. Some scholars have studied the method of improving GAP, and the optimized strength is generally achieved. For example, the strength of the film prepared by Xu and Ma et al. reached 1.5 MPa and 1.6 MPa, and the elongation reached 81.6%. Compared with the existing test results, the strength and elongation of the GAP film reported in this paper are much higher than the existing results [11,14,16].

### 3.4. The SEM of Tensile Cross-Section of Energetic Polymer Composites

**Figure 5:**
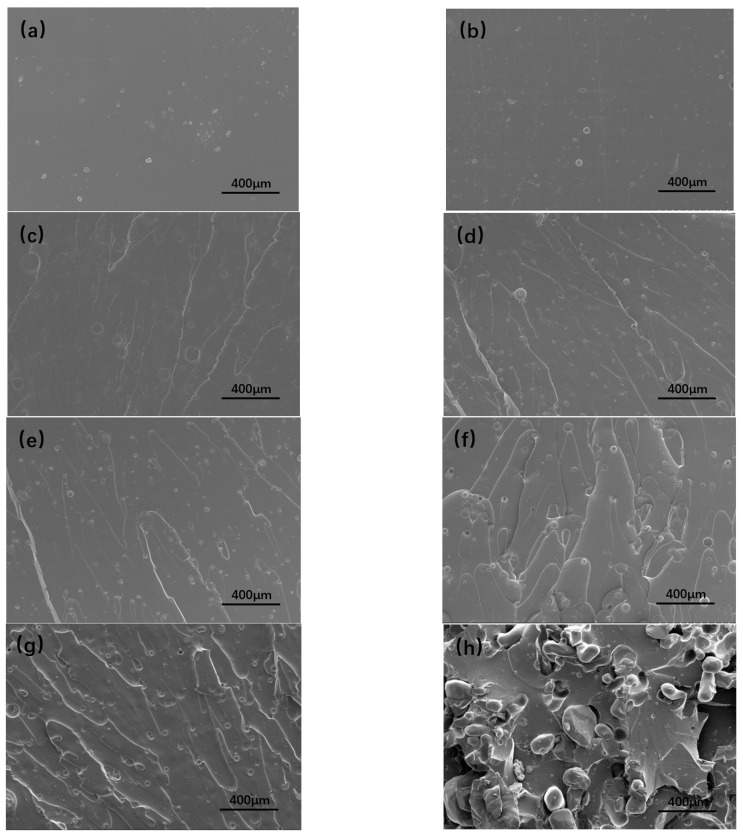
The SEM of tensile cross-section of polymer films with different R = 1.2 (**a**), 1.4 (**b**), 1.6 (**c**), 1.8 (**d**), 2.0 (**e**), 2.2 (**f**), 2.4 (**g**), and energetic polymer composites with 40% RDX with R = 2.2 (**h**).

The SEM images of tensile cross-section of the polymer films with different R were shown in Figure 5a–g. With R increases, the ‘circles’ appear in the section, due to the degree of phase separation in the polymer film increasing and the stripes after tensile fracture becoming more obvious. The overall sections were relatively smooth, indicating that the films obviously had brittle fractures under tensile action. The number of folds in the section increased with the increase of R, and the actual surface area of the fracture surface increased, so that a large amount of energy was dissipated through the generation of new surface.

In order to understand the dispersion of RDX in the adhesive system, the fracture surface of energetic polymer composite with 40% RDX was observed by SEM (Figure 5h). The tensile fracture surface was rougher, and the particle distribution was more uniform. However, the RDX particles were separated from the adhesive system, and some holes appeared. These holes were formed by the RDX particles distributed in the films falling off the fracture surface under the action of external force. Increasingly obvious folds appeared around the holes and particles, indicating that the RDX particles combined with the matrix can be used as stress concentration sites to transfer stress and dissipate energy, thereby reducing the crack growth rate.

### 3.5. Mechanical Property of Energetic Polymer Composites with Different RDX Contents

**Figure 6:**
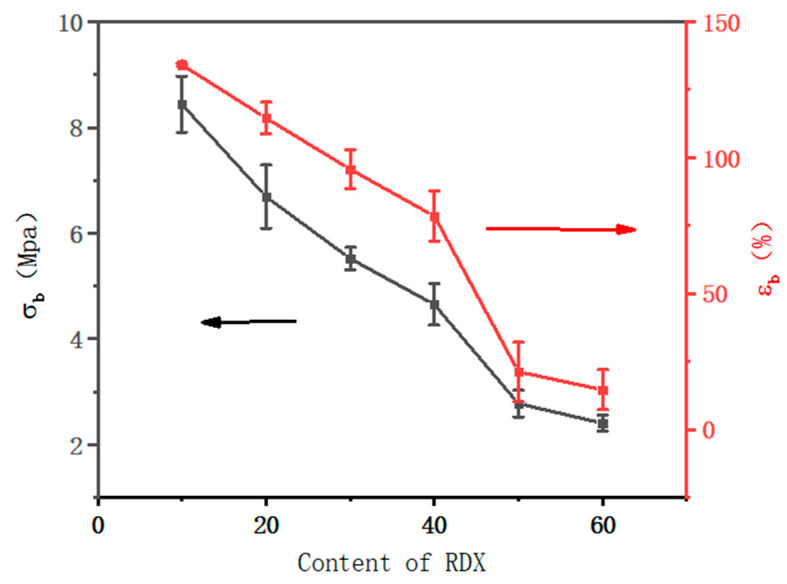
The curves of tensile strength and elongation at break of energetic polymer composites with different RDX contents.

The mechanical properties of energetic polymer composites with different RDX particle contents were tested. The mechanical tensile and elongation at the breaks of polymer composites are shown in Figure 6. When the content of RDX particles is increased from 10 wt.% to 60 wt.%, the mechanical strength of polymer composites decreased from 8.44 MPa to 2.41 MPa, and the elongation at break decreased from 134.18% to 14.60%. This reason is attributed to two factors: one is that with the increases of RDX particle content, the content of polymer adhesive decreases; the other is that there is a poor interfacial binding force between polymer adhesive and RDX particles; these particles cannot constrain the movement of polymer chains, leading to a decrease in mechanical properties.

### 3.6. The Heat of Explosive and Residual Carbon Rate of Energetic Polymer Composites with Different RDX Contents

To study key properties of energetic polymer composites, the heat of explosive and residual carbon rate with different RDX contents was tested—the results are shown in Table 4. It was observed that with the increase of RDX contents, the heat of the explosive of polymer composites increased, and residual carbon rate decreased, indicating that polymer composite with 60 wt.% RDX perform well.

## 4. Conclusions

Energetic polymer composites were prepared with different R consisting of GAP used as the adhesive, TDI, and glycerol and its composites. With the increase of R, mechanical strength gradually increased from 0.43 MPa to 19.58 MPa, and elongation at break decreased from 337.51% to 94.98%. When the content of RDX particles increases with R = 2.2, the mechanical tensile and elongation at break of polymer composites both decrease from 8.44 MPa to 2.41 MPa, and from 134.18% to 14.60%, respectively. In addition, when the loading of the RDX content is 60 wt.% and R = 2.2, the values of the heat of explosive and residue carbon rate of polymer composites are 3.29 MJ/kg and 1.78%, respectively. GAP as a clean adhesive has been widely studied in aerospace, polymers, and other fields, as it is environmentally friendly. This work provides support for the research on improving the mechanical properties of eco-friendly energetic polymer composites.

## Figures and Tables

**Table 1:** Feeding ratio of polymer films with different R.

R	GAP/%	TDI/%	GLY/%
1.2	89.71	8.74	1.03
1.4	87.03	11.16	1.31
1.6	83.81	14.04	1.65
1.8	79.88	17.56	2.06
2.0	74.95	21.97	2.58
2.2	68.60	27.65	3.25
2.4	60.12	35.24	4.14

**Table 2:** The bonded carbonyl ratio of GAP/TDI/GLY polymer films with different R.

R	Free-Carbonyl	Bonded-Carbonyl	Total-Carbonyl	Bonded-Carbonyl-Ratio (%)
1.2	2.0771	2.0006	4.0778	49.06
1.4	3.0331	3.1961	6.2291	51.31
1.6	9.9886	3.9343	6.9228	56.83
1.8	1.5847	2.1949	3.7796	58.07
2.0	0.6030	1.0923	1.6953	64.43
2.2	1.2901	2.3794	3.6695	64.84
2.4	8.8463	9.7253	16.572	58.69

**Table 3:** Crosslink density and correction factor of polymer films with different R.

R	ν_e_ × 10^−4^ (mol/cm^−3^)	ρ (g/cm^−3^)	E (MPa)	Mc (kg/cm^−3^)	A
1.2	1.115	1.299	0.055	1.165	−0.235
1.4	1.231	1.297	0.126	1.054	−0.237
1.6	1.302	1.295	0.226	0.995	−0.240
1.8	4.823	1.291	2.196	0.268	−0.361
2.0	8.033	1.288	6.709	0.160	0.409
2.2	11.06	1.283	17.001	0.116	3.157
2.4	12.84	1.277	71.228	0.099	20.815

**Table 4:** The heat of explosive and residue carbon rate of energetic polymer composites with different RDX contents.

RDX Content (%)	The Heat of Explosive (MJ/kg)	Residue Carbon Rate (%)
10	2.25	6.75
20	2.41	5.28
30	2.51	4.09
40	2.87	2.47
50	3.09	2.20
60	3.29	1.78

## Data Availability

The data presented in this study are available on request from the corresponding author.
